# Cellular Kinetics of Perivascular MSC Precursors

**DOI:** 10.1155/2013/983059

**Published:** 2013-08-19

**Authors:** William C. W. Chen, Tea Soon Park, Iain R. Murray, Ludovic Zimmerlin, Lorenza Lazzari, Johnny Huard, Bruno Péault

**Affiliations:** ^1^Stem Cell Research Center, Department of Orthopaedic Surgery, School of Medicine, University of Pittsburgh, Pittsburgh, PA 15219, USA; ^2^Department of Bioengineering, University of Pittsburgh, Pittsburgh, PA 15260, USA; ^3^Institute for Cell Engineering and Department of Pediatric Oncology, School of Medicine, Johns Hopkins University, Baltimore, MD 21205, USA; ^4^Centre for Regenerative Medicine, University of Edinburgh, Edinburgh, EH16 4TJ, UK; ^5^Orthopaedic Hospital Research Center and David Geffen School of Medicine at UCLA, University of California at Los Angeles, 615 Charles E. Young Drive South, Los Angeles, CA 90095-7358, USA; ^6^Cell Factory, Fondazione Ospedale Maggiore Policlinico, 20122 Milan, Italy; ^7^McGowan Institute for Regenerative Medicine, Pittsburgh, PA 15219, USA; ^8^Centre for Cardiovascular Science, University of Edinburgh, Queen's Medical Research Institute, 47 Little France Crescent, Edinburgh EH16 4TJ, UK

## Abstract

Mesenchymal stem/stromal cells (MSCs) and MSC-like multipotent stem/progenitor cells have been widely investigated for regenerative medicine and deemed promising in clinical applications. In order to further improve MSC-based stem cell therapeutics, it is important to understand the cellular kinetics and functional roles of MSCs in the dynamic regenerative processes. However, due to the heterogeneous nature of typical MSC cultures, their native identity and anatomical localization in the body have remained unclear, making it difficult to decipher the existence of distinct cell subsets within the MSC entity. Recent studies have shown that several blood-vessel-derived precursor cell populations, purified by flow cytometry from multiple human organs, give rise to *bona fide* MSCs, suggesting that the vasculature serves as a systemic reservoir of MSC-like stem/progenitor cells. Using individually purified MSC-like precursor cell subsets, we and other researchers have been able to investigate the differential phenotypes and regenerative capacities of these contributing cellular constituents in the MSC pool. In this review, we will discuss the identification and characterization of perivascular MSC precursors, including pericytes and adventitial cells, and focus on their cellular kinetics: cell adhesion, migration, engraftment, homing, and intercellular cross-talk during tissue repair and regeneration.

## 1. Introduction

The availability of mesenchymal stem/stromal cells (MSCs) and MSC-like multipotent stem/progenitor cells marked a major milestone in stem cell therapies [1, 2]. For more than a decade, MSC has been a highly promising stem cell source and extensively investigated for its therapeutic potentials [3, 4]. Unlike embryonic stem cells (ESCs) or induced pluripotent stem cells (iPSCs), MSCs are inherently more relevant to clinical applications due to the lack of ethical and safety issues, despite lower developmental versatility [5]. MSCs and similar mesodermal stem/progenitor cells have been shown to repair and/or regenerate a wide variety of damaged/defective organs, including bone, cartilage, muscle, heart, and skin [6–10]. MSCs have also been reported to support hematopoiesis and suppress immune reaction after cell/organ transplantation [11–14].

Nevertheless, owing to the nature of MSC isolation by plastic adherence in tissue culture, the native identity and anatomical localization of MSCs have remained unclear for years [15]. Recently, several studies have indicated that MSCs represent a heterogeneous entity in culture, and a number of multipotent precursor cells potentially contributing to the MSC pool have been identified *in vivo* [16, 17]. Increasing evidence further suggests that MSCs and some tissue-specific progenitor cells are anatomically and functionally associated with vascular/perivascular niches in various tissues [18–21]. Following the hypothesis that blood vessels throughout the body serve as a systemic reservoir of multipotent stem/progenitor cells, we and other researchers have identified, purified, and characterized distinct populations of MSC-like multilineage precursors from the vasculature of multiple human organs [17, 22]. These human blood vessel-derived precursor cell subsets, including pericytes (PCs) [23], adventitial cells (ACs) [24], and myogenic endothelial cells (MECs) [25], can be isolated via fluorescence-activated cell sorting (FACS) based on their unique expression of cell surface antigens. Purified PCs, ACs, and MECs not only exhibit typical mesodermal multipotency in culture but also demonstrate robust regenerative capacities in animal disease models. Consequently these precursor cell subsets, particularly PCs and ACs that can be universally derived from definitive structures of blood vessel walls, represent active contributors to the MSC entity [17].

In this review, we will discuss the identification and characterization of perivascular MSC precursors (i.e., PCs and ACs) from multiple organs and focus on their cellular kinetics during regenerative events, including cell adhesion, migration, engraftment, homing, and intercellular cross-talk.

## 2. Native Distribution of MSCs and MSC-Like Multipotent Stem/Progenitor Cells

MSCs and MSC-like stem/progenitor cells have been found in nearly all organs in the human body. Despite slight differences in phenotypes and cellular functions, MSCs and MSC-like cells from various ontogenies share basic features in general, including selective plastic adherence, expression of typical MSC surface markers, and mesenchymal multipotency such as osteogenesis, chondrogenesis, and adipogenesis. Some of the most common MSCs and MSC-like multilineage cells are briefly introduced here. 

### 2.1. Bone Marrow-Derived MSCs (BM-MSCs)

Bone marrow (BM) harbors multiple types of stem/progenitor cells, including hematopoietic stem cells (HSCs), endothelial progenitor cells (EPCs), and BM-MSCs [26, 27]. As a standard MSC population, BM-MSCs are defined as nonhematopoietic, plastic adherent progenitor cells that self-renew, differentiate into typical mesodermal cell lineages including osteogenic, chondrogenic, and adipogenic lineages, and express CD73, CD90, and CD105 but are negative for CD11b, CD14, CD19, CD34, CD45, CD79*α*, and HLA-DR1 [28]. Estimated by the colony forming unit fibroblasts assay (CFU-F) *in vitro,* BM-MSCs typically exist at a very low frequency within the BM mononucleated cell population (0.01%–0.1% of total BM cells) but can be efficiently expanded in culture, making them one of the most investigated autologous stem/progenitor cell populations. Interestingly, multipotent BM-MSC clones retain approximately twofold higher CD146 expression level than unipotent clones [29]. 

### 2.2. Adipose-Derived Stem/Stromal Cells (ASCs)

The stromal vascular fraction (SVF) of adipose can be isolated via enzymatic digestion of intact fat tissue or lipoaspirate, followed by the depletion of mature adipocytes through centrifugation. The SVF embodies a broad and heterogeneous cellular compartment, including vascular cells (endothelial and perivascular populations), hematopoietic cells (resident and circulating cells), and stromal fibroblasts. In 1976, human adipogenic progenitors (aka preadipocytes) were successfully isolated by two independent groups from the adipose SVF by selective adherence to culture plastics [30, 31]. The adherent fraction of the adipose SVF was later identified as a source of mesenchymogenic progenitors [32], termed adipose-derived stem/stromal cells (ASC) [33]. ASCs are defined *in vitro* using the same criteria as *bona fide* BM-MSCs [34], including their selective plastic adherence, mesenchymal differentiation capacities and immunophenotypes [32], although ASCs only resemble BM-MSCs at subsequent passages in culture [35]. Unlike BM-MSCs, early-passage ASCs temporarily retain expression of mucosialin (CD34) [35], a well-established marker for stem/progenitor cells in both hematopoietic [36] and endothelial [37] cell lineages. On another note, the temporary retention of CD34 expression in primary ASCs led to confusion regarding their origin *in situ*. This misperception was accentuated in light of the recent characterization of CD34-negative PCs as a source of MSCs in a variety of mesodermal tissues, including fat [23]. While the adipogenic activity is mainly exhibited by the prevalent CD34+/CD31− subset of the adipose SVF [38], the CD34-negative fraction can also generate ASCs *in vitro* [24, 39, 40]. Immunohistochemical studies have confined these mesenchymogenic subpopulations to the adipose microvasculature where they coexist, respectively, in the media and adventitia in an annular fashion [24, 39, 41, 42]. Both PCs and an outer supra-adventitial layer of CD34-positive cells (adventitial cells/supra-adventitial stromal cells, ACs) possess high adipogenic potential *in vitro* [39, 43] and may contribute together to replenish the pool of adipocytes essential to sustain the high fat turnover* in vivo* [44]. 

### 2.3. Umbilical Cord-Derived Mesenchymal Stem/Stromal Cells (UC-MSCs)

Stem/progenitor cells isolated from disposable perinatal tissues, including amnion/amniotic fluid, umbilical cord blood, placental tissue, umbilical cord blood vessels, and the Wharton's jelly, have been deemed promising for clinical applications because of the minimal safety and ethical concerns [45, 46]. MSCs and MSC-like cells have been isolated from different compartments of the umbilical cord, including umbilical vein subendothelial zone, umbilical cord blood, and specifically, Wharton's jelly [45, 47]. Wharton's jelly is the parenchyma within the umbilical cord, a mucoid connective tissue surrounding umbilical cord arteries and vein [45]. The Wharton's jelly can be further divided into three anatomical regions where MSCs can be derived from the perivascular zone, the intervascular zone, and the subamnion [47]. Similar to BM-MSCs, MSCs derived from Wharton's jelly exhibit plastic adherence, mesenchymal multipotency, and expression of CD10, CD13, CD29, CD44, CD73, CD90, CD105, and HLA-class I but are negative for CD11b, CD14, CD19, CD31, CD34, CD45, CD56, CD79, and HLA class II [45–47].

## 3. Blood Vessels as a Source of MSC Precursors

The similarities between MSCs derived from many different tissues aroused the idea that a common reservoir of MSCs may exist in the body. The blood vessel, which typically consists of three structural layers: *tunica intima*, *tunica media*, and *tunica adventitia* [48], is distributed throughout nearly all human organs and therefore represents a favorable candidate. Early evidence supporting the hypothesis that the vascular wall serves as a systemic source of stem cells came from a study of the emerging hematopoietic system in the embryo and fetus, where hematopoietic cells emerged in close vicinity to vascular endothelial cells (ECs) in both intra- and extraembryonic blood-forming tissues [22]. Recently, several studies have indicated the possibility that blood vessels in different organs contain multilineage precursors that possess MSC-like features and contribute to tissue repair/regeneration [49, 50]. New evidence further pointed out that tissue-specific multipotent stem/progenitor cells, including osteogenic, neural, odontoblastic, and adipogenic progenitors, may originate from and/or associate with vascular/perivascular niches *in vivo *[18–21]. 

Microvascular pericytes (PCs), a set of perivascular mural cells surrounding *the intima* of microvessels and capillaries, are traditionally regarded as a structural component of blood vessels, regulating vascular contractility, stability, and integrity [51, 52]. Intimate interactions between PCs and ECs tightly regulate vascular growth, maturation, and remodeling [51, 53–55]. Recently, PCs have been implicated in a number of pathological conditions, making them potential targets for therapeutic interventions [55, 56]. On the other hand, the *tunica adventitia*, the outermost layer of large blood vessels, has long been considered as a structural bystander, consisting of loosely structured collagen-rich extracellular matrix (ECM), which embeds stromal cells/fibroblasts, the *vasa vasorum*, and perivascular nerves [57]. The importance of the *tunica adventitia* was recently reevaluated due to a number of studies reporting its active role in vascular remodeling, immune response mediation, cell trafficking, and atherosclerosis [57–59]. In a vascular remodeling setting following an injury, it has been shown that adventitial cells (ACs) start a process of proliferation, migration into the tunicae media and intima, and differentiation into smooth muscle cells [60–62]. Recently, we and several other groups reported new strategies for the identification and purification of the elusive PCs and ACs [23, 24, 39, 63–65]. Using immunohistochemistry and flow cytometry, we identified human PCs and ACs *in situ* and purified these cells to homogeneity based on their unique expressions of cell surface antigens. Details of the isolation and characterization of PCs and ACs will be described in the following sections.

Unlike the *tunicae media* and *adventitia*, the subendothelial zone of *tunica intima* has previously been suggested as one of the sources of EPCs [66, 67]. Apart from PCs and ACs, some of us have also reported a rare but distinct subset of blood-vessel-derived stem cells, that is, myogenic endothelial cells (MECs), residing within the intima of microvasculature in human skeletal muscle [25]. MECs, presumably the human counterpart of murine muscle derived stem cells (MDSCs), not only express the myogenic cell marker, CD56, but also display endothelial cell markers, CD34 and CD144. Following purification by FACS, MECs (CD34+/CD56+/CD144+/CD45−) can be clonally expanded and exhibited osteo-, chondro-, adipo-, and myogenic differentiation capacities *in vitro* [25]. Furthermore, MECs exhibited superior cardiac repair capacity in ischemic hearts and myogenic regeneration in injured skeletal muscle than conventional CD56+ myoblasts and ECs [25, 68, 69]. Nevertheless, despite their MSC-like features and tissue reparative/regenerative capability, whether MECs contribute significantly to the MSC entity remains to be clarified due to their restricted presence in skeletal muscle.

## 4. Identification and Purification of Perivascular MSC Precursors

### 4.1. Placenta

While placenta and umbilical cord are often discarded at birth, these extraembryonic tissues contain large numbers of stem/progenitor cells, making them attractive sources of donor cells for regenerative medicine. We and others have isolated multipotent PCs (CD146+/CD34−/CD45−/CD56−) from these tissues and utilized them toward multiple tissue repair/regeneration, including skeletal muscle [70], lung [71], dermal [72], and nervous tissues [73]. 

**Figure 1:**
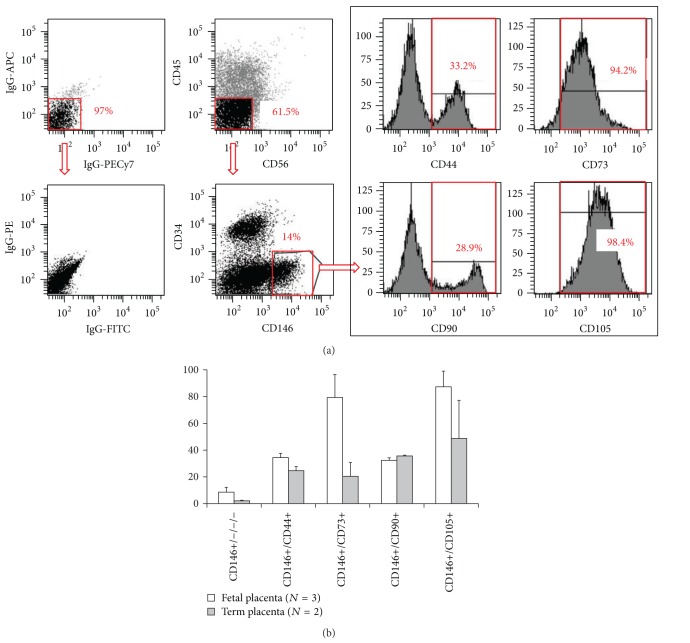
Flow cytometry analysis of mesenchymal stem cell marker expression in freshly isolated fetal and term placental pericytes. (a) Representative flow cytometry analysis of human placenta that was mechanically dissociated and enzymatically digested and subsequently stained for CD45, CD56, CD34, and CD146 along with CD44, CD73, CD90, or CD105. Matching isotype controls were shown in the left column. (b) Human fetal placenta (*N* = 3, average 20 weeks of gestation) and term placenta (*N* = 2, average 39 weeks of gestation) were used to isolate subsets of pericytes using surface expression of CD146+/CD34−/CD45−/CD56− (CD146+/−/−/−) and colabeled with one of the mesenchymal stem cell markers (CD146+/CD44+, CD146+/CD73+, CD146+/CD90+, CD146+/CD105+) as shown in (a). Values are mean ± standard error.

Placenta is a highly vascularized extraembryonic tissue, which serves as fetomaternal interface to sustain proper oxygen transportation, waste disposal, and nutrient delivery. The placental vasculature has been thoroughly characterized throughout fetal development previously and consists of all sizes/types of blood vessels and both pericytes/perivascular cells and ECs at all stages [74, 75]. Placenta PCs are critical to maintain blood vessel homeostasis and promote angiogenesis [76, 77]. PC abnormity in placenta capillaries leads to defects in sinusoidal integrity, a phenotype often observed during pregnancy complications due to diabetes, postmaturity, or preeclampsia [78]. In addition to their supportive role in the fetal vasculature, placental PCs have also been identified as a source of MSCs [23, 70, 79]. Our previous studies have discriminated mesenchymogenic placental PCs based on the expression of the cell adhesion molecule CD146 and lack of EC markers: CD34, CD144, and vWF [23, 70]. Similarly, Castrechini et al. described a perivascular population residing in human fetal and term placenta, which coexpressed MSC/PC markers (Stro-1, 3G5, CD105, CD106, CD146, CD49a, *α*-SMA) but not hematovascular markers (CD117, CD34, vWF) and were competent for trilineage mesenchymal differentiation [79]. In our hands, human fetal and term chorionic villi of placentas included 8.5 ± 3.66% (*N* = 3, 19 to 21 weeks of gestation) and 2.1 ± 0.43% (*N* = 2, 39 weeks of gestation) of PCs (CD146+/CD34−/CD45−/CD56−), respectively (Figure 1).

The native expression of CD146 by mesenchymogenic PCs in many tissues including bone marrow, fetal and term placentas has been reported [23, 70]. Using FACS, we purified PCs from mechanically and enzymatically dissociated placental chorionic villi [23, 70]. Freshly isolated placenta PCs natively expressed MSC markers (CD44, CD73, CD90, and CD105) at varying levels (30 to 87% of fetal and 20 to 48% of term placental CD146+/CD34−/CD45−/CD56− PCs) (Figure 1). We have previously demonstrated that when placed onto ECM-coated plates, dissected fetal placental villi release a population of vascular cells, which possess high migratory activity and robust capacity to regenerate skeletal muscle fibers in dystrophic mice [70]. The cells migrating out of placental villi included predominantly CD146+ cells which coexpressed PC (NG2 and PDGFR*β*) and MSC (CD44, CD73, CD90, and CD105) surface antigens and were deprived of EC antigens (CD31, CD34, CD144, and vWF) [70]. Maier et al. employed a similar approach to isolate PCs from the cellular outgrowth of human term placenta explants [80]. Consistently with fetal placenta, term placenta PCs expressed high levels of PC/MSC markers (CD146, PDGFR*β*, NG2, CD90, and calponin), including 65 transcripts that are highly expressed in undifferentiated MSCs, and lacked endothelial/hematopoietic cell marker expression (CD31, CD34, and CD45) [80]. 

### 4.2. Umbilical Cord

Human umbilical cord (HUC) has been known as an abundant source of ECs as well as MSCs derived from the Wharton's jelly. Recently some of us demonstrated that human full-term UCs and, at a higher frequency, fetal (preterm) UCs contain perivascular cells that exhibit features of MSCs. These perivascular smooth muscle-like cells present in the HUC co-expressed CD146 and alpha-smooth muscle actin (*α*SMA) but did not express the established EC markers: CD144, CD34, CD31, and Ulex europaeus agglutinin (UEA-1) receptor. Using FACS, Montemurro et al. isolated a population of PCs (CD146+/NG2+/PDGFR*β*+) from umbilical cords of preterm newborns [71]. These HUC-derived perivascular cells (HUCPCs) can be maintained in long-term culture, exhibiting classical spindle-shape PC morphology. When characterized by flow cytometry during subsequent passages, they maintained the expression of CD44, CD90, CD73, CD105, HLA class I, CD146, NG2, *α*SMA, and PDGFR*β* as well as retained their multipotency to differentiate towards different cell types, including osteogenic, adipogenic, and myogenic cell lineages [71].

### 4.3. Skeletal Muscle

Skeletal muscle has been shown to harbor several adult stem/progenitor cell populations in mammals including humans, in addition to the typical muscle stem cells, that is, satellite cells [81–83]. Many studies have demonstrated that muscle derived stem/progenitor cells are capable of differentiating into a variety of cell lineages *in vitro* and *in vivo*, including blood cells and fat [25, 81, 84–86]. Using similar immunohistochemical and flow cytometry strategies, we first identified microvascular PCs *in situ* within human skeletal muscle and subsequently purified them from mechanically and enzymatically dissociated muscle biopsies via FACS [23]. Similar to PCs sorted from other tissues, muscle PCs (CD146+/CD34−/CD45−/CD56−) expressed typical PC markers: CD146, NG2, PDGFR-*β*, alkaline phosphatase (ALP), and *α*-smooth muscle actin (*α*-SMA), with the absence of EC markers: CD31, CD34, CD144, and vWF as well as the hematopoietic cell marker CD45 and myogenic cell marker CD56. Muscle PCs can be efficiently expanded in culture, at the clonal level, while maintaining robust mesodermal developmental potentials. Freshly isolated and long-term cultured muscle PCs both displayed robust myogenic capacity *in vitro *and *in vivo*. Moreover, muscle PCs natively and in culture expressed classic MSC markers: CD44, CD73, CD90, and CD105, indicating their developmental status as MSC ancestors [23]. 

### 4.4. Adipose

Vasculogenic CD34+/CD31− cell populations have been described in the adventitial *vasa vasorum* of large blood vessels such as the vena saphena [65] and the thoracic aorta [67], but microvascular CD34+ ACs seem to be a specific feature of the adipose and subcutaneous tissue [87]. Apart from CD34 expression and their adjacent anatomical localization within the blood vessel wall, ACs can be discriminated from adipose PCs due to the lack of native expression of PC markers (*α*SMA, CD146, NG2, PDGFR*β*) [24, 39, 42]. The high prevalence (*∼*50%) of CD34+/CD146− progenitor cells in the nonhematopoietic adipose SVF [39, 88, 89] and their limited clonogenicity and heterogeneous proliferative capacity [24] do not preclude the possibility that distinct CD34+ stem/progenitor cells exist within adult adipose tissue. Using a peroxisome proliferator-activated receptor gamma (PPAR*γ*) reporter mouse model, Tang et al. demonstrated that adipogenic progenitors emerge from CD34+ cells which later adopt a perivascular niche and express PC markers (*α*SMA, NG2, PDGFR*β*) [21]. Similarly, human adipose CD34+/CD146− ACs can acquire PC markers (*α*SMA, CD146, NG2, PDGFR*β*) *in vitro*, following treatment with angiotensin II or angiopoietin-2 [24]. 

While developmentally mesenchymogenic PCs may arise from transient CD34+ cell population(s), the persistence of such CD34+ precursors in the adult and their ontological relationship to the bulk of CD34+ ACs in human fat will require further investigation. Indeed, rare CD34+ mesenchymogenic cells have been reported in fetal [24, 90, 91] and adult [92, 93] bone marrow, as well as in fetal muscle and fetal lung [24]. A multipotent CD34+ cell population residing in the wall of dorsal aorta, the mesoangioblast, has been proposed to be an ancestor of adult mesenchymogenic PCs in the mouse [49, 81]. Some groups have reported the direct derivation of CD34+ primitive MSCs from human embryonic stem cells (hESC) [94, 95], while Vodyanik et al. described the emergence of a multipotent MSC precursor, the mesenchymoangioblast, from hESC-derived CD34+ cells in a stepwise differentiation system [96]. Furthermore, Dar et al. recently reported successful derivation of CD105+/CD90+/CD73+/CD31− multipotent mesodermal precursors from embryoid bodies of either human ESCs or iPSCs that exhibit clonogenicity, mesenchymal differentiation potentials, and* bona fide* pericyte features, including angiogenic/vasculogenic capacity and expression of CD146, NG2, and PDGFR*β* but not *α*SMA, CD56, CD34, or EC markers [97]. These hPSC-derived PCs significantly facilitated vascular and muscle regeneration when transplanted into the ischemic limb of immunodeficient mice, with the presence of hPSC-PCs in both recovered vasculature and myofibers, indicating robust vasculogenic and myogenic capacities *in vivo* similar to their adult counterparts [97]. Yet, the reciprocity of all these fetal populations to all or part of adult MSC precursors remains to be clarified. 

A rare CD34+/CD146+/CD31−/CD45− population of adipose PCs has also been characterized in the SVF [39, 98–103] and may represent a developmental intermediate between PCs and some or all ACs [102]. This elusive CD34+ PC population is not easily detected within the vascular wall by immunohistochemistry [24, 42] and requires stringent rare-event strategies for its detection and isolation by flow cytometry [100, 103]. Traktuev et al. suggested the existence of CD34+ cells exhibiting a native pericytic phenotype [98]. They demonstrated that primary cultures of AC-like CD34+CD144−CD45− SVF cells can express PC markers (NG2, PDGFR*α*, PDGFR*β*) without requirement of blood vessel remodeling growth factors in contrast to CD34+CD146− cells [24]. Though these disparities may be related to culture conditions, SVF isolation techniques, and cell sorting strategies, the intricacy and anatomical proximity of these distinct subpopulations highlight the necessity to use multidimensional strategies for their isolation via exclusion of hematopoietic (CD45) and endothelial (CD31, CD144) lineages and combinatory positive selection of pericytic (i.e., CD146, NG2, PDGFR*β*), adventitial (CD34), or MSC (CD44, CD73, CD90, CD105) cell subsets. A number of studies have employed preliminary sorting strategies relying on single markers, such as CD146 [104, 105] or CD34 [40, 106, 107], which may be inadequate in regard to the overlapping phenotypes of the vascular/perivascular cell subsets populating the adipose tissue. 

Recently, using a combination of above-mentioned positive and negative selection antigens, we performed advanced flow cytometry analyses and FACS in the adipose SVF in order to identify and simultaneously purify these MSC precursor subpopulations [23, 24, 39, 101]. Both CD146+/CD34−/CD45− PCs and CD34+/CD31−/CD45−/CD146− ACs purified from adipose SVF have been shown to express MSC markers *in vivo* and in culture [23, 24, 101]. Furthermore, our quantitative multiparameter studies showed that only a third of adipose PCs (CD146+/CD34−/CD31−/Lineage−/CD45−) natively coexpress the MSC markers CD73, CD90, and CD105, which reveals the cellular heterogeneity of the pericyte compartment [101]. In contrast, both CD146+ (putative PC-AC intermediates) and CD146− (ACs) subsets of CD34+/CD31−/Lineage−/CD45− SVF cells homogenously co-express MSC markers [101]. On the other hand, among these MSC-like perivascular cells, two subpopulations in the adipose SVF can be discerned on the basis of CD34 expression and further distinguished by their proliferation pattern: a low proliferative CD34− subset and a high proliferative CD34+ subset. While CD34− is a typical phenotype of multipotent mesenchymogenic PCs in adipose and most other tissues [23], the CD34+ phenotype may represent transit-amplifying intermediates between stem-like adipose PCs and highly prevalent ACs *in vivo* but require prudent interpretations in culture due to its instability.

## 5. Adhesion and Migration of Perivascular MSC Precursors

In view of future stem cell-based approaches and therapies, it is crucial to identify predictive parameters that allow the researchers and clinicians to foresee the *in vivo* action of stem/progenitor cells. Since cell adhesion and migration capacities are tightly correlated with *in vivo* cell trafficking and homing, these parameters represent potential predictors for the clinical outcome of stem cell-treated patients and require further investigation [108–110]. Herein we discuss recent progresses in the understanding of perivascular MSC precursors in regard to cell adhesion, migration, and response to hypoxia.

### 5.1. Cell Adhesion

Anatomically, PCs closely surround ECs populating the vascular intima with specific adhesion and migration properties that allow them to regulate the blood vessel stability/integrity as well as the proliferation and motility of adjacent ECs [51]. Up to 1000 contacts can be secured by peg-sockets to a juxtaposing EC via cytoplasmic fingers inserted into endothelial invaginations [111]. Pericytic elongated terminal arms include adhesion plaques that strongly embed into the basement membrane and EC body to secure their location [111]. Different molecules and pathways have been involved in mural cell motility and adhesion. Notably, ephB/ephrin-B interactions mediate human MSC/PC adhesion, migration, and differentiation [112, 113]. The eph/ephrin family of tyrosine kinase receptors has been identified as an important factor contributing to bone homeostasis and regulating MSC adhesion. Inhibition of ephrin-B signaling prevents MSC attachment and spreading by activation of Src-, PI3 Kinase-, and JNK-dependent signaling pathways [112]. Ephrin-B2-deficient mural cells display major defects in spreading, focal-adhesion formation, and polarized migration as well as exhibiting increased motility [113]. Our group investigated adhesion molecules and proteins involved in PC migratory capacity. We demonstrated that CD146+/NG2+/PDGFR*β*+/CD144− PCs exhibited more robust adherence to extracellular matrix substrates (e.g., collagen type-I, gelatin, and fibronectin) and greater migratory capacity than the CD146− population. Enhanced adherence and migratory capacities may result from high expression levels of alpha and beta subunits of integrin and matrix metalloproteinase (MMP)-2, respectively [70]. On the other hand, PCs express intercellular adhesion molecule 1 (ICAM-1) and upregulate its expression in response to tumor necrosis factor (TNF) and pattern-recognition receptor (PRR) ligands. ICAM-1 also regulates interactions of neutrophils and monocytes with PCs *in vitro *[114]. Moreover, it has been suggested that arteriolar and capillary PCs can detect inflammatory stimuli and increase their adhesive interactions with innate leukocytes, implicating their role in the regulation of inflammatory responses [114, 115].

### 5.2. Cell Migration

PC recruitment and migration occur frequently in response to pathophysiological events such as wound healing, inflammation, or angiogenesis. During vascular development, ECs release PDGF-BB to recruit PCs and stabilize the newly formed blood vessels [116, 117]. Increase of PC density by activation of PDGF-BB/ PDGFR*β* signaling pathways has also been detected during wound healing and tumor vascular remodeling [56, 111, 118]. Inversely, disruption of PDGF-BB/PDGFR*β* pathways may occur during pathologic conditions (e.g., diabetic retinopathy), resulting in PC apoptosis and augmented permeability of the vascular wall [111, 119]. Upon inflammatory events, PCs control the pattern and efficiency of leukocyte interstitial migration *in vivo* [114, 120]. A recent study highlighted the constitutive expression of chemoattractants by NG2+ PCs: CSC-chemokine ligand-1 (CXCL1) and -8 (CXCL8), macrophage migration inhibitory factor (MIF), CC-chemokine ligand 2 (CCL2), and interleukin-6 (IL-6). PCs further upregulated the expression of these chemo-attractants following stimulation by PRR ligands [114, 115]. Therefore, PCs not only chemotactically migrate to the site of angiogenesis, injury, or inflammation but also actively recruit other proinflammatory participants, including myeloid leukocytes, neutrophils, and macrophages.

Using an *in vitro* model of tissue damage, some of us previously mimicked the ability of HUCPCs to migrate towards the injury site *in vivo* and predicted their capacity to secrete cytokines and trophic factors [71]. Envisioning a possible clinical application of stem cells in the context of extremely immature newborns with an acute lung injury, where alveolar type II cells crucial for producing surfactant and regulating alveolar fluid levels and host defense are damaged, HUC can be readily considered as a convenient source of stem cells. Consequently, a coculture model of pulmonary tissue damage was set up, where an alveolar type II cell line was damaged with bleomycin, an anticancer drug with known pulmonary toxicity [71]. Dye-labeled HUCPCs in coculture were mobilized and migrated towards the damaged alveolar type II cells. HUCPCs showed a great ability to secrete angiogenic/antiapoptotic cytokines and trophic factors compared to the control, in particular high level of keratinocyte growth factor (KGF) [71]. KGF appears to play a crucial role mediating tissue improvement in a range of experimental lung injuries, presumably due to its versatile effects including cellular repair, cytoprotection, and alveolar fluid clearance modulation and immunomodulation [121, 122]. Similarly, skeletal muscle-derived PCs secrete high levels (superior to those of BM-MSCs) of KGF and vascular endothelial growth factor (VEGF) as well as heparin binding-epidermal growth factor (HB-EGF) and basic-fibroblast growth factor (bFGF), which are all considered playing critical roles during wound healing [123, 124].

The abundance of mesenchymogenic progenitors in the SVF of adipose tissue (5,000 CFU-F per gram) [125] provides a great advantage for the development of clinical applications without any *in vitro* expansion requirements [126, 127]. ASC-based therapeutic strategies have been proposed for either regenerative or targeted therapies and often rely on native tropism of ASCs for wound healing, inflammation, or cancer. Although investigations of cell adhesion and migration in purified ACs are currently ongoing, much can be learned from the unfractionated ASCs which have been shown to home to sites of injury and promote tissue repair following systemic injections in animal models of myocardial infarction [128, 129], liver injury [130, 131], olfactory dysfunction [132], hypoxia-ischemia induced brain damage [133], allergic rhinitis [134], inflammatory neuropathy [135], sciatic crush [136], cranial injury [137], and muscular dystrophy [138, 139]. The migratory activity of early-passage ASCs can be modulated by a set of chemokines and growth factors, including PDGF-AB, TGF-*β*1, and TNF*α* [140]. These soluble factors can stimulate ASCs via activation of an array of migration-associated receptors such as C-C chemokine receptor types 1 and 7 (CCR1, CCR7), C-X-C chemokine receptor types 4, 5, and 6 (CXCR4, CXCR5, CXCR6), EGF receptor, fibroblast growth factor receptor 1, TGF-*β* receptor 2, TNF receptor superfamily member 1A, and PDGF receptors *α* and *β* [140–142].

ASCs have been proposed to affect various neighboring cells within the subcutaneous tissue via paracrine signals during active remodeling processes such as wound healing [143–145]. In a recent study, ASC-conditioned medium promoted *in vitro* migration of vascular ECs, fibroblasts, and keratinocytes [146]. These data support the impact of ASCs on the proliferation and recruitment of these distinct cell subsets during wound healing via secretion of high levels of promigratory cytokines, including angiopoietin-like-1, EGF, FGF, HGF, TGF*β*, SDF-1, and VEGF [145–149]. 

Similarly to BM-MSCs [150, 151], ASCs have been associated with enhanced migratory activities during tumorigenesis. ASC tropism towards various tumors such as glioma [152, 153], colon cancer [154], and prostate cancer [155] has been exploited to develop targeted therapies. On the other hand, ASCs can modulate the migration of cancer cells, promoting metastasis of breast cancer cells [156, 157] via CCR5/CCL5 signaling in animal models despite the inhibition of breast cancer metastasis in a different model [158]. An antimetastatic result was also observed with pancreas cancer cells [159]. 

### 5.3. Cellular Response to Hypoxia

Hypoxia has been shown to promote proliferation and migration of both PCs and MSCs [160, 161]. A recent study highlighted the involvement of the ERK signaling pathway during the modulation of mitogenic and chemotactic responses of human muscle PCs to a low oxygen concentration (6% O_2_). This activation of ERK signaling and associated integrins occurred without any detectable alteration on the cell phenotypes or differentiation potentials [160, 162]. A number of growth factors, including PDGF, EGF, and FGF, can activate the Ras-Raf-MEK1/2-ERK signaling axis [163], which controls the adhesion dynamics and cell migratory properties via formation of protrusions within cell membrane and enhancement of the focal adhesion turnover [164]. Culture of MSCs in hypoxic conditions also resulted in higher survival and migration in a hind-limb ischemia model, presumably through Akt signaling [165]. The activation of the Akt pathway has been linked to the cell migratory ability and can be mediated by hepatocyte growth factor (HGF). MSCs under hypoxia exhibited higher expression of cMet, a critical HGF receptor [165, 166], and two receptors of the chemokine stromal-derived factor-1 (SDF-1), CXCR4 and CXCR7, whose expression can also be mediated by hypoxia via the hypoxia-inducible factor-1 alpha (HIF-1*α*) and Akt phosphorylation [167]. Additionally, even under a 2.5% O_2_ hypoxia, the paracrine function of PCs remained highly active when compared to 21% O_2_ normoxic culture, with increased expression of VEGF-A, PDGF-B, and TGF*β*1 and decreased expression of angiopoietin-1, bFGF, EGF, HGF, and MCP-1, and similar levels of leukemia inhibitory factor (LIF), cyclooxygenase-2 (COX-2/PTGS-2, prostaglandin endoperoxide synthase-2), heme oxygenase-1 (HMOX-1), IL-6, HIF-1*α*, and MMP-2 [168]. Understanding cellular responses of perivascular MSC precursors and MSCs to hypoxia would help researchers and clinicians to develop better approaches to improve the efficacy of MSC-based cell therapy, including genetic modification, cellular preconditioning, and pharmacological adjunct therapy [9]. 

## 6. Migratory and Homing Characteristics of Perivascular MSC Precursors during Tissue Repair/Regeneration

Perivascular MSC precursors have recently been demonstrated as efficient regenerative/supportive units for tissue repair and regeneration. In particular, human muscle PCs and saphenous vein-derived ACs exhibited superior angiogenic, paracrine, and cardioprotective capacities and augmented functional recovery in murine myocardial infarction and hind-limb ischemia models when compared to myoblasts or unfractionated MSCs [65, 168, 169]. Additionally, muscle and placental PCs were shown to repair/regenerate injured and dystrophic muscles in animal disease models as well as contribute to the muscle stem cell (satellite cell) pool [23, 64, 70, 170]. Some of us also showed that HUCPCs prevented/rescued the oxygen-induced arrest in alveolar growth and restored lung function and architecture, primarily through their paracrine function [171]. Interestingly, CD146+ PCs extracted from adipose tissue were shown to support the long-term persistence of human hematopoietic stem/progenitor cells in coculture [172]. Moreover, purified human PCs and ACs exhibited bone formation or healing when implanted into animal models of ectopic bone formation or critical-sized calvarial bone injury, respectively [88, 89, 173]. In this section, we will discuss the current understanding of the cell engraftment, migration, and homing of transplanted perivascular MSC precursors during some of these regenerative events.

### 6.1. Cardiac Repair

**Figure 2:**
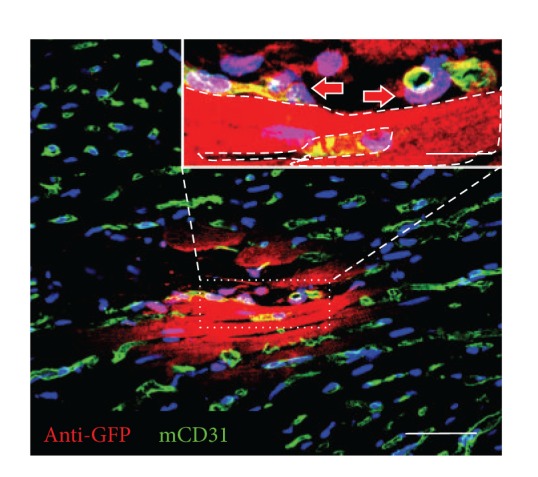
Human pericytes home to perivascular locations. Confocal microscopy showed that GFP+ human pericytes (red), identified by anti-GFP immunostaining, can be located at the interstitial space where host CD31+ capillaries (green) reside (main, scale bar = 50 *μ*m). Some GFP+ donor cells (inset, red arrows) are in close contact with mouse CD31+ endothelial cells (green). Dash line in the inset picture delineates a putative GFP+ cardiomyocyte (inset, scale bar = 10 *μ*m).

When intramyocardially transplanted into a mouse model of acute myocardial infarction (AMI), purified human muscle PCs contributed to cardiac functional and anatomic recovery after infarction, presumably through multiple cardioprotective and regenerative mechanisms: reversal of ventricular remodeling, reduction of cardiac fibrosis, diminution of chronic inflammation, promotion of host angiogenesis, and small-scale myocardial regenerative events [168]. The engraftment ratio of intramyocardially injected GFP-labeled PCs was approximately 9% at the first week, decreasing to roughly 3% at 8 weeks after infarction. Above all, a fraction of donor PCs was identified in perivascular positions, juxtaposing host CD31+ ECs (Figure 2). In contrast to the engraftment ratio, the vessel-homing ratio of transplanted PCs slightly increased over time, implicating the potential benefit of niche-homing for long-term donor cell survival. Moreover, cellular interactions between donor PCs and host ECs were demonstrated by the expression of human-specific ephrin type-B receptor 2 (EphB2) in some GFP+ PCs adjacent to ECs as well as the formation of connexin 43 gap junctions with ECs [168]. Additionally, immune cells in the ischemic tissue release chemokines such as interleukins and monocyte chemoattractant protein-1 (MCP-1), which are involved in the homing of MSCs to the ischemic heart [174]. Moreover, the paracrine anti-inflammatory function of human MSCs was also demonstrated by the high expression of anti-inflammatory protein TSG-6 from MSCs embolized in lung, which led to decreased inflammatory responses, reduced infarct size, and improved cardiac function [175].

Similarly, Katare et al. reported that transplantation of human saphenous vein-derived ACs (hSV-ACs), a putative PC progenitor population, promoted functional improvement in a mouse model of MI, primarily through angiocrine activities and neovascularization via both donor and recipient cells as well as other cardioprotective mechanisms including improved myocardial blood flow, attenuated vascular permeability, and reduction of myocardial remodeling, cardiomyocyte apoptosis, and interstitial fibrosis [169]. hSV-ACs produced and released microRNA-132 (miR-132) as a paracrine agent, which exerts proangiogenic, prosurvival, and antifibrotic activities and likely plays a key role as an activator of cardiac healing. While retaining their original antigenic and perivascular phenotype, homing of hSV-ACs to perivascular locations was confirmed by Dil-labeled hSV-ACs juxtaposing isolectin-positive capillary ECs [169]. 

### 6.2. Muscle Regeneration

As mentioned previously, we have demonstrated that intramuscular injection of freshly sorted or cultured PCs derived from human adipose or skeletal muscle regenerated human myofibers efficiently in the mouse dystrophic or injured muscle [23]. In another study, we showed that intramuscular implantation of dissected human placental villi resulted in crude outgrowth of human cells in dystrophic mice [70]. Ample amount of cells of human origin released from placental villi fragments participated in host muscle regeneration, revealed by the detection of human dystrophin-positive (hDys3t) and/or human spectrin-positive myofibers. Many of these human myofibers coexpressed human lamin A/C, indicating their sole human origin and not intermediate products of cell fusion. Surprisingly, human myofibers were located not only close to the implantation area (500 *μ*m to 2 mm) but also in far more distant regions (up to 2 cm), suggesting active migration of outgrown human myogenic precursors over long distances. Similarly, freshly isolated placental PCs possessed high migratory activity and actively contributed to host skeletal muscle regeneration [70]. 

### 6.3. Pulmonary Repair

As mentioned previously, PCs isolated from umbilical cords migrated efficiently *in vitro* toward alveolar type II cells damaged by bleomycin, with an elevated secretion of KGF and VEGF [71]. Using a preclinical animal model of oxygen-arrested lung growth (exposure to 95% oxygen, i.e., hyperoxia), which mimics bronchopulmonary dysplasia (BPD), Pierro et al. tested the *in vivo* therapeutic potential of HUCPCs [171]. To examine suitable approaches for future clinical applications, two different administration strategies, prophylactic or therapeutic, as well as two different therapeutic modalities, direct cell transplantation or HUCPC-conditioned medium injection, were investigated. Intratracheal transplantation of HUCPCs prevented/rescued oxygen-induced arrested alveolar growth and restored normal alveolar architecture. However, immunofluorescence and qPCR revealed very few donor cells localized within the lung. This low cell engraftment suggested that cell replacement is not the primary mechanism of the observed therapeutic effects. Indeed similar therapeutic benefits can be achieved by daily intraperitoneal administration of conditioned medium, resulting in improved alveolar architecture and lung function. In both administration strategies, long-term efficacy and safety were demonstrated till 6 months with an improved exercise capacity and normal alveolar architecture. No suspicious tumor formation was noted by total body CT scans. In conclusion, the therapeutic potential of HUCPCs for pulmonary repair can be exploited by either direct cell therapy or the production of trophic factors, expanding new clinical perspectives for HUCPCs and other perivascular MSC precursors.

### 6.4. Skeletal Regeneration

To investigate their skeletal regenerative capacity, human PCs and ACs purified from lipoaspirate SVF have been seeded onto osteoinductive scaffolds and implanted into animal models of ectopic bone formation or critical-sized calvarial bone injury, respectively [88, 89, 176]. Significantly greater osteogenesis or bone healing by PCs and ACs in murine muscle pockets or calvarial defects than control SVF cells was observed, respectively. Additionally, the high osteogenic capability of human ACs and PCs can be further enhanced by Nel-like molecule-1 (NELL-1), an osteoinductive growth factor that is a direct transcriptional target of Runx2 [89, 173, 176, 177]. On the other hand, the role of the SDF-1/CXCR4 pathway in MSCs/PCs recruitment during the injury response has been established in a murine model of femoral bone graft, where SDF-1 deficient mice were unable to recruit MSCs at bone fracture sites and consequently limited their participation to local bone repair [178]. The role of the SDF-1/CXCR4 axis in PC recruitment has also been revealed during tumorigenesis [179]. Overexpression of PDGF-BB increased malignant PC growth via activation of the SDF-1/CXCR4 axis and induced expression of SDF-1 in ECs. The upregulation of SDF-1 was directly mediated by inhibition of the Akt/mTOR pathway or HIF-1*α*. Accordingly, both donor and host stem cell homing can be further enhanced by MSCs genetically modified to overexpress SDF-1 [180]. 

## 7. Angiogenic Capacities of Perivascular MSC Precursors and Cellular Interactions with ECs

### 7.1. Pericyte-EC Cellular Interactions: A Perivascular Niche for MSC Precursors

PCs are ubiquitously present in microvasculature where they extend primary cytoplasmic processes along the abluminal surface of the endothelial tube. They are enveloped in a basement membrane that is continuous with the EC basement membrane to which both cells contribute [181, 182]. The majority of the PC-EC interface is separated by basement membrane, with the two cell types contacting each other at discrete points through peg-socket type interactions, occluding contacts, gap junctions, and adhesion plaques [183, 184]. The intimate anatomical relationship between ECs and PCs suggests close interactions involving not only direct contact but also paracrine or juxtacrine signaling. EC-to-PC ratios in normal tissues vary between 1 : 1 to 10 : 1 and may be up to 100 : 1 (in skeletal muscle), while PC coverage of the endothelial abluminal surface ranges between 10% and 70% [185, 186]. PC density and coverage appear to correlate with endothelial barrier properties (i.e., brain > lungs > muscle) [111], EC turnover (large turnover leading to less coverage) [184], and orthostatic blood pressure (larger coverage in lower body parts) [185], in keeping with a role of PCs in regulating capillary barriers, endothelial proliferation, and capillary diameter [111]. Genetically modified mouse models have demonstrated that these two vascular cell types are interdependent: primary defects in one cell type have obligated consequences for the other. There is growing evidence to suggest that ECs can manipulate the migratory and angiogenic properties of PCs, while *in vitro *data highlighting EC influence on mesenchymal differentiation potential of PCs points to a possible role of ECs as gatekeepers within the context of an adult stem cell niche.

### 7.2. EC Interactions Regulate Pericyte Recruitment and Angiogenesis

**Figure 3:**
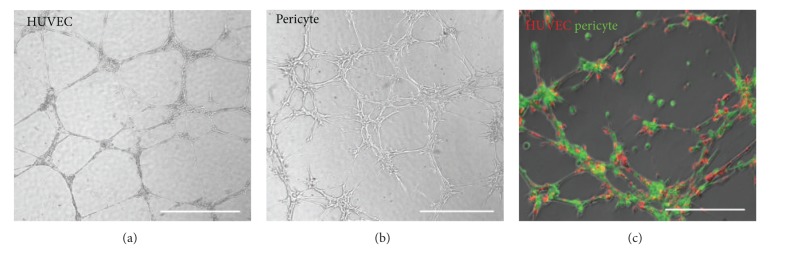
Human pericytes support formation of microvascular structures. (a) HUVECs seeded onto Matrigel-coated wells formed typical capillary-like structures after 24 hours (scale bar = 1 mm). (b) Human muscle pericytes formed morphologically similar network structures within 6–8 hours (scale bar = 1 mm). (c) Cocultured dye-labeled HUVECs (red) and pericytes (green) at 1 : 1 ratio on Matrigel showed coformation of capillary-like networks within 6–8 hours (scale bars = 500 *μ*m).

The formation of new capillaries during angiogenesis requires a series of well-orchestrated cellular events allowing ECs and PCs to migrate into the perivascular space. In vessel sprouting, angiogenic factors (e.g., VEGF) stimulate ECs, which in turn secrete proteases that degrade basement membrane and allow EC invasion. An endothelial column, guided by a migrating EC at the very tip, then moves toward a VEGF gradient [183]. Studies of the *corpus luteum* indicate that PCs are also capable of guiding sprouting processes by migrating ahead of ECs and expressing VEGF [198–200]. Emerging endothelial tubes then secrete growth factors, partly to attract PCs that envelop the vessel wall, and promote vessel maturation. Key pathways implicated in PC-EC signaling include PDGF/PDGFR*β*, angiopoietins and Tie receptors, sphingosine-1-phosphate signaling, TGF-*β* signaling, Notch and Wnt [116, 186, 201, 202]. It is believed that PCs, because of their vessel-embracing position, are able to transfer angiogenic signals along the vessel length by contacting numerous ECs. The recruitment and contribution of PCs to developing endothelial tubes and angiogenic process can be observed *in vitro* through Matrigel culture. Human muscle PCs alone can form network structures in Matrigel culture that were morphologically similar to networks formed by ECs but at an accelerated fashion (Figures 3(a) and 3(b)). Coculture of dye-labeled PCs and ECs at 1 : 1 ratio in Matrigel showed network formation by both cell types, facilitated by the presence of PCs (Figure 3(c)). Blocki et al. further demonstrated that while the capacity to colocalize and/or coform network structures with endothelial tubules on Matrigel is not restricted to PCs, only PCs (CD146+/CD34−) effectively stabilize endothelial networks and improve endothelial sprout integrity [203]. Nevertheless, it is noteworthy that the EC-to-PC ratio may play an important role in the formation of vascular networks and PC functionality *in vitro*.

### 7.3. ECs: The Gatekeepers of Pericyte Mesenchymal Activation?

A growing number of studies demonstrate that tissue resident stem cells reside in vascular niches, including neural, hematopoietic, and MSCs [19, 204–206]. Adult stem cell niche components provide signals that control the balance between quiescence, self-renewal, and differentiation [205]. A significant obstacle in identification of the perivascular origin of MSCs was the reluctance of PCs to express mesenchymal phenotypes in their native microenvironment [207]. Although it is feasible that PCs acquire MSC potentials upon exiting the microvasculature, it is intuitive that MSC features are expressed by PCs *in situ* but environmentally downregulated. Studies using unfractionated SVF have demonstrated poor and unreliable tissue formation [208] or lower regeneration efficacy relative to prospectively isolated and purified MSCs [208], lending further support to a hypothesis that certain cellular component(s) of SVF have an inhibitory/adverse effect on differentiating MSCs. As such, the influence of ECs on the multipotency of tissue-specific MSCs is now under investigation even though preliminary results to date have been divergent (Table 1). Osteogenic and adipogenic differentiation is not seen within the perivasculature of healthy tissues where the PC-EC relationship is undisturbed. However, disturbed PC-EC interactions have been observed in conditions associated with pathological mineralization and adipogenesis, for example, heterotopic ossification and atherosclerosis [209, 210]. In addition, the ECM proteins, also present within a perivascular niche, have been shown to modify growth and differentiation of MSCs, with collagen type I-, fibronectin-, and vitronectin-treated plates enhancing mineralization *in vitro* [211]. The secretome and proteome of human MSCs have now been extensively documented [212] with studies identifying numerous transcription factors and multiple extracellular and intracellular signaling pathways that regulate adipogenesis and osteogenesis. Interestingly, inducers of differentiation along one lineage often inhibit differentiation along another. For example, the transcription factor PPAR*γ* is a prime inducer of adipogenesis that inhibits osteogenesis, highlighting the mutual exclusivity of these lineages [213]. It is therefore likely that signaling mechanisms responsible for the mesenchymal fate of PCs will be multifactorial and distinct for different lineages. 

## 8. Conclusion

In this review, we described the identification and characterization of perivascular MSC precursors with regard to their adhesion, migration, engraftment/homing, and intercellular cross-talk in culture and in experimental animal models. Although PCs and ACs both exhibit multilineage mesenchymogenic capacities and are derived from adjacent perivascular structural layers, further investigations are required to clarify their developmental relationship as well as the involvement of an ontogenic intermediate. Through the understanding of their unique cellular kinetics and regenerative potential, we will be able to define the pathophysiological role and therapeutic value of the individual blood-vessel-derived MSC precursor population under a particular pathological circumstance. Ultimately, through the purification and/or recombination of these distinct subsets of MSC precursors, it is feasible to further enhance stem cell therapy by eliminating cells with none or limited regenerative potentials in a specific disorder, creating a customized therapeutic modality for the personalized medicine.

## Figures and Tables

**Table 1:** The influence of ECs on the multipotency of tissue-specific MSCs.

Niche Component	Model	Stem cell surrogate	Niche surrogate	Lineage assessed	Effect on differentiation	Context	Proposed mechanism	Investigator
Endothelial cell	3D	ASC	HUVEC	Osteogenesis	↓	Paracrine	↑Wnt	Rajashekhar et al. [187]
Endothelial cell	3D	ASC	HUVEC	Osteogenesis	↓	Juxtacrine	↑Wnt	Rajashekhar et al. [187]
Endothelial cell	2D	BMSC	HUVEC	Osteogenesis	↑	Paracrine	(Dkk1-Wnt, FGF, PDGF, BMP, TGF*β*, Notch)	Saleh et al. [188]
Endothelial cell	2D	BMSC	HUVEC	Adipogenesis	—	Paracrine	—	Saleh et al. [189]
Endothelial cell	2D	BMSC	HUVEC	Osteogenesis	↑	Juxtacrine	—	Xue et al. [190]
Endothelial cell	2D	BMSC	HDMEC	Osteogenesis	↑	Juxtacrine	BMP-2	Kaigler et al. [191]
Endothelial cell	2D	BMSC	HDMEC	osteogenesis	—	Paracrine	—	Kaigler et al. [191]
Endothelial cell	2D	BMSC	HDMEC	Osteogenesis	↑	Juxtacrine	N-cadherin	Li et al. [192]
Endothelial cell	2D	BMSC	HDMEC	Osteogenesis	↑	Paracrine	VEGF	Grellier et al. [193]
Endothelial cell	2D	BMSC	HDMEC	Osteogenesis	↓	Paracrine	Osterix/OSX	Meury et al. [194]
Endothelial cell	2D	BMSC	HUVEC	Osteogenesis	↑	Juxtacrine	Cx43/gap junctions	Villars et al. [195]
Endothelial cell	2D	BMSC	HUVEC	Osteogenesis	↑	Juxtacrine	—	Villars et al. [196]
Endothelial cell	2D	HOP	HUVEC	Osteogenesis	↑	Juxtacrine	—	Guillotin et al. [197]
Endothelial cell	2D	HOP	EPC, HSVEC	Osteogenesis	↑	Juxtacrine	Cx43/gap junctions	Guillotin et al. [197]
